# GluN2B-containing NMDARs in the mammalian brain: pharmacology, physiology, and pathology

**DOI:** 10.3389/fnmol.2023.1190324

**Published:** 2023-05-31

**Authors:** Yang Ge, Yu Tian Wang

**Affiliations:** ^1^Djavad Mowafaghian Centre for Brain Health, University of British Columbia, Vancouver, BC, Canada; ^2^Department of Medicine, University of British Columbia, Vancouver, BC, Canada

**Keywords:** NMDAR (NMDA receptor), GluN2B (NMDA receptor subunit NR2B), synaptic plasticity (LTP/LTD), neuronal death, neurological disorders

## Abstract

Glutamate N-methyl-D-aspartate receptor (NMDAR) is critical for promoting physiological synaptic plasticity and neuronal viability. As a major subpopulation of the NMDAR, the GluN2B subunit-containing NMDARs have distinct pharmacological properties, physiological functions, and pathological relevance to neurological diseases compared with other NMDAR subtypes. In mature neurons, GluN2B-containing NMDARs are likely expressed as both diheteromeric and triheteromeric receptors, though the functional importance of each subpopulation has yet to be disentangled. Moreover, the C-terminal region of the GluN2B subunit forms structural complexes with multiple intracellular signaling proteins. These protein complexes play critical roles in both activity-dependent synaptic plasticity and neuronal survival and death signaling, thus serving as the molecular substrates underlying multiple physiological functions. Accordingly, dysregulation of GluN2B-containing NMDARs and/or their downstream signaling pathways has been implicated in neurological diseases, and various strategies to reverse these deficits have been investigated. In this article, we provide an overview of GluN2B-containing NMDAR pharmacology and its key physiological functions, highlighting the importance of this receptor subtype during both health and disease states.

## Introduction

The N-methyl-D-aspartate glutamate receptor (NMDAR) has been widely recognized as a key family of ligand-gated glutamate receptors (Paoletti et al., 2013). Although the NMDAR channel mediates postsynaptic depolarization in response to the excitatory neurotransmitter glutamate, it generally contributes little to the fast synaptic transmission, but mediates more complex physiological functions in the central nervous system (CNS; Paoletti et al., 2013). One of the central themes related to NMDAR function is that the receptors play a critical role in the bidirectional, activity-dependent changes of synaptic strength, which is known as synaptic plasticity (Collingridge et al., 2004; Paoletti et al., 2013). A second theme related to NMDAR function is that these receptors mediate neuronal survival and death during neuronal development and neuronal excitotoxicity, a delayed neuronal death process in response to the overexcitation of neurons (Tashiro et al., 2006; Wu and Tymianski, 2018; Ge et al., 2020). Based on the importance of neuronal plasticity and the regulation of neuronal survival and death during both physiological and pathological conditions, understanding the precise physiological functions of NMDARs is critical for discovering the fundamental processes related to learning and memory and the pathological mechanisms underlying multiple neurological diseases.

The diversity of NMDAR subunits plays a central role in mediating the complex functions of NMDARs (Paoletti et al., 2013). Genome sequencing experiments have first identified multiple NMDAR subunits, which are capable of assembling into various functional NMDAR subtypes, as demonstrated by subsequent biological and electrophysiological characterizations (Monyer et al., 1994; Sheng et al., 1994). More recently, the functional diversity of NMDAR subunits has been increasingly acknowledged, facilitated by the availability of pharmacological tools and genetic mice models to achieve targeted manipulation of each of the several different NMDAR subtypes (Liu et al., 2004; Brigman et al., 2010; Paoletti et al., 2013). These studies have identified NMDARs containing the GluN2B subunit as, arguably, the most widely expressed subtypes in forebrain neurons and the most important NMDAR subtype contributing to neuronal development, synaptic plasticity, and neuronal excitotoxicity (Sheng et al., 1994; Paoletti et al., 2013; Ge et al., 2020). Here, we will summarize the current understanding of the pharmacology, physiology, and pathology related to GluN2B-containing NMDARs with a focus on the more recent investigations.

## Pharmacology of GluN2B-containing NMDARs

### Subunit composition

**Figure 1:**
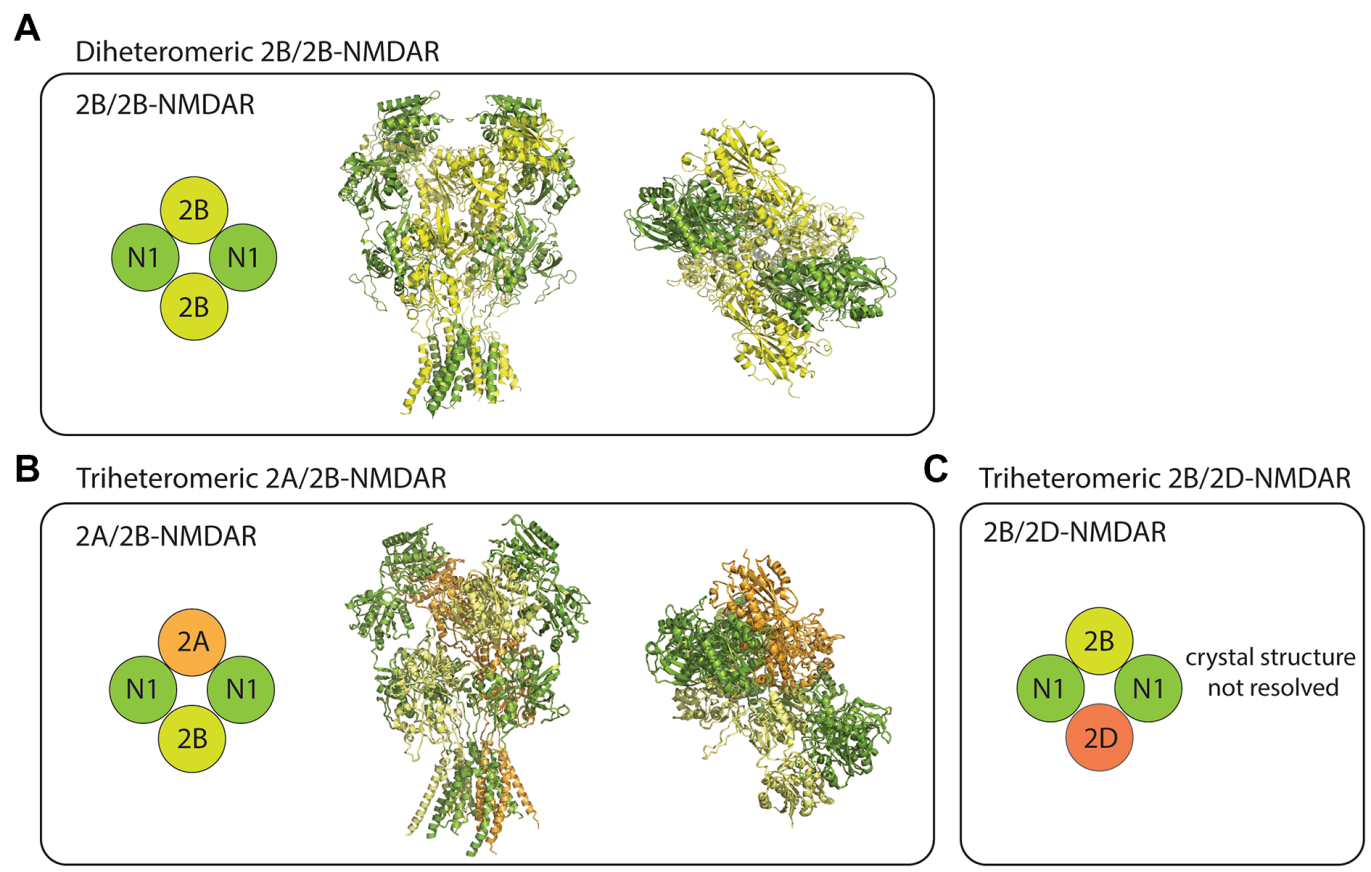
Subtype diversity and structures of GluN2B-containing NMDARs. **(A)** The diheteromeric GluN2B/GluN2B (2B/2B)-NMDAR is composed of two GluN1 (N1) and two GluN2B (2B) subunits (left). Sideview (middle) and topview (right) of 2B/2B-NMDAR structure resolved from x-ray diffraction (PDB: 4PE5). **(B)** The triheteromeric GluN2A/GluN2B (2A/2B)-NMDAR is composed of two N1, one GluN2A (2A), and one 2B subunits (left). Sideview (middle) and topview (right) of 2A/2B-NMDAR structure resolved from electron microscopy (PDB: 5UOW). **(C)** The triheteromeric GluN2B/GluN2D (2B/2D)-NMDAR has two N1, one 2B, and one GluN2D (2D) subunits. Crystal structures were visualized using PyMOL academic version.

The NMDARs are heterotetrameric complexes composed of eight different subunits in humans (and in rodents) including GluN1, GluN2(A-D), and GluN3(A/B; Collingridge et al., 2004). While the two GluN1 subunits are essential for the formation of functional NMDAR complexes, the remaining GluN2/3 subunits determine the receptor’s functional diversity (Paoletti et al., 2013). In the case of GluN2B-containing NMDARs, at least three different compositions have been studied using recombinant expression systems. These receptor subtypes include the diheteromeric GluN1/GluN2B (2B/2B)- (Hansen et al., 2014; Yi et al., 2019), triheteromeric GluN1/GluN2A/GluN2B (2A/2B)- (Hansen et al., 2014), and triheteromeric GluN1/GluN2B/GluN2D (2B/2D)-NMDARs (Yi et al., 2019; Figure 1). Despite some recent advancements in the pharmacological understanding of each receptor subtype, the proportion of diheteromeric and triheteromeric GluN2B-containing NMDARs expressed in CNS neurons is still a subject of debate.

Although triheteromeric GluN2B-containing NMDARs may be an important subpopulation of GluN2B-containing NMDARs in CNS neurons, most physiological studies overlook their contribution, primarily due to the inability of currently used experimental techniques to separate them from diheteromeric 2B/2B-NMDARs. To further complicate this issue, when used at their typical working concentrations, subtype-specific antagonists that are known to almost fully block the diheteromeric 2B/2B-NMDAR currents generally block a proportion (~30%–60%) of the triheteromeric GluN2B-containing NMDARs currents (Hansen et al., 2014; Stroebel et al., 2018). Thus, reduction of NMDAR function by GluN2B-specific antagonists could theoretically be due to either a full blockade of diheteromeric 2B/2B-NMDAR function or a partial blockade of triheteromeric GluN2B-containing NMDAR function. Nevertheless, accumulating evidence supports that at least some diheteromeric 2B/2B-NMDARs are present throughout the life span of neurons, and they have channel functions and downstream signaling properties that are different from non-GluN2B-containing NMDARs. Consequently, the physiological functions that will be discussed in this review are thought to be contributed largely by the diheteromeric 2B/2B-NMDARs. It is important to acknowledge, however, that the same physiological functions may be contributed by triheteromeric GluN2B-containing NMDARs.

### Temporospatial expression

The GluN2B subunit shows distinct temporospatial expression patterns from other GluN2/3 subunits (Wenzel et al., 1997). Sharp contrasts have been drawn between the expression patterns of the GluN2B and GluN2A subunits. For example, the GluN2B subunit is by far the most expressed GluN2/3 subunit in embryonic murine brains; however, its expression peaks at around the first postnatal week, after which it is partially and gradually replaced by the increasingly expressed GluN2A subunit (Wenzel et al., 1997). In adult murine forebrain neurons, one commonly cited finding is that the GluN2B subunit is mainly located extrasynaptically, whereas the GluN2A subunit is primarily expressed at the neuronal synapses (Stocca and Vicini, 1998). Nevertheless, this model of subunit-dependent localization of NMDAR subtypes is likely too simplistic, since some studies have reported a substantial proportion of GluN2B-containing NMDAR-mediated synaptic currents in more mature neurons (Thomas et al., 2006; Pegasiou et al., 2020).

The temporospatial expression of diheteromeric vs. triheteromeric GluN2B-containing NMDAR subtypes is still a subject of immense debate. Based on the predominant expression of the GluN2B subunit during early postnatal days, it is reasonable to hypothesize that diheteromeric 2B/2B-NMDAR is the dominant NMDAR complex during early development (Wenzel et al., 1997). As the expression of the GluN2A subunit increases (particularly at the postsynaptic density), synaptic NMDARs in the cortical neurons may start to be populated by diheteromeric 2A/2A-or triheteromeric 2A/2B-NMDARs (Paoletti et al., 2013). Nevertheless, the exact composition of the NMDARs throughout the developmental time course has yet to be determined by using more sophisticated structural biology methods, such as using cryogenic electron microscopy (cryo-EM) to investigate the subunit stoichiometry of native NMDARs in the CNS (Zhao et al., 2019).

### Subtype-specific pharmacological modulators

Some divalent cations, including Zn^2+^ and Mg^2+^, modulate the activities of NMDARs in a subtype-specific manner (Nowak et al., 1984; Paoletti et al., 2013). For example, Zn^2+^ is a more potent allosteric inhibitor on diheteromeric 2A/2A-NMDARs than diheteromeric 2B/2B-NMDARs, and, thus, a frequently used pharmacological tool for investigating GluN2A-containing NMDAR functions (Nozaki et al., 2011); however, its specificity for NMDARs containing the GluN2A subunit is limited by its similarly potent inhibition on triheteromeric 2A/2B-NMDAR currents and its modest efficacy of blocking (~50%) diheteromeric 2A/2A-NMDAR currents under physiological pH (Hansen et al., 2014). NMDAR subtypes also experience voltage-dependent channel blocking by Mg^2+^, and the Mg^2+^ sensitivity is roughly 10 times stronger for NMDARs containing the GluN2A/GluN2B subunits compared with those containing the GluN2C/GluN2D subunits (Paoletti et al., 2013). Importantly, the relief from Mg^2+^ inhibition of the GluN2A and/or GluN2B-containing NMDAR currents during repetitive or strong synaptic activities is critical for activity-dependent synaptic plasticity (Paoletti et al., 2013). The subtype-dependent modulation of the activities of GluN2B-containing and other NMDAR subtypes, by these divalent cations, are important physiological mechanisms for regulating neuronal functions.

Developing subtype-specific modulators for GluN2B-containing NMDARs has been a long-standing interest for both research and therapeutic purposes. Multiple GluN2B-containing NMDAR antagonists have been tested in preclinical studies and in clinical trials, most of which are analogous to a phenylethanolamine called ifenprodil, which binds to a GluN1-GluN2B subunit interface at the N-terminal domain (Karakas et al., 2011; Liu W. et al., 2020). Some of the antagonists in this class, such as Ro25-6981 and CP-101606, have achieved outstanding subtype specificity with over 1,000-fold higher affinity for diheteromeric GluN2B-containing NMDARs compared with other diheteromeric NMDAR subtypes (Chenard et al., 1995; Fischer et al., 1997). Although Ro25-6981, when applied at its working concentration, also blocks a proportion of the currents measured from recombinantly expressed triheteromeric 2A/2B-NMDARs (Hansen et al., 2014), its blocking effect on the 2A/2B-NMDAR currents is small (~30%) compared with that on diheteromeric 2B/2B-NMDAR currents (>90%). Therefore, this drug has been a useful tool for investigating the physiological functions of diheteromeric 2B/2B-NMDARs.

Several endogenous polyamines in the CNS, including spermine and spermidine, positively modulate the GluN2B-containing NMDAR currents (Williams et al., 1994). Importantly, the positive modulation is specific to the diheteromeric 2B/2B-NMDARs with little to no effect on other NMDAR subtypes, including the triheteromeric 2A/2B-NMDARs (Stroebel et al., 2014). It is important to note that polyamines generally have low potency toward modulating the diheteromeric 2B/2B-NMDAR currents (EC50 around hundreds of μM) and may also affect many other targets in the CNS at the concentration that is commonly used (Guerra et al., 2016). Further understanding of the structural biology of polyamine modulation on diheteromeric NMDARs, including the resolution of a crystal structure illustrating the binding of polyamines to 2B/2B-NMDARs, may facilitate the development of more potent and specific modulators to investigate the functions of diheteromeric 2B/2B-NMDARs.

## Physiological functions of GluN2B-containing NMDARs

It has been widely recognized that GluN2B-containing NMDARs often have distinct physiological functions compared with other NMDAR subtypes. Genetic knockout/knockin and pharmacological inhibition have been the most important approaches for studying the functions of GluN2B-containing NMDARs, and a wide range of physiological functions has been investigated (Paoletti et al., 2013). While a comprehensive discussion of GluN2B-containing NMDAR functions is out of the scope of this article, we will focus on the roles of this receptor subpopulation on synaptic plasticity and neuronal fate (particularly in terms of neuronal survival and death).

### Roles of GluN2B-containing NMDAR in LTP

Long-term potentiation (LTP) has been acknowledged as a well-investigated form of activity-dependent synaptic plasticity in mammalian brains. Although the roles of GluN2B-containing NMDARs in LTP may be ambivalent overall, many earlier lines of evidence support the importance of this NMDAR subgroup in the induction of LTP (Shipton and Paulsen, 2014). First, compared with the GluN2A subunit, the GluN2B subunit has a much higher affinity for calcium calmodulin-dependent kinase II (CaMKII), a key protein kinase that is involved in LTP induction (Barria and Malinow, 2005). Knockin mice with the GluN2B subunit’s CaMKII binding site residues replaced by the analogous ones on the GluN2A subunit have severely impaired LTP (Barria and Malinow, 2005). Furthermore, genetic ablation of the GluN2B subunit in the hippocampal CA1 region abolishes LTP in a subset of hippocampal circuits, whereas transgenic mice overexpressing the GluN2B subunit have dramatically enhanced LTP in the hippocampal CA3-CA1 synapse (Tang et al., 1999; Akashi et al., 2009). Together, these data support the hypothesis that GluN2B-containing NMDARs and their associated protein kinases play essential roles in the induction of LTP.

Many recent studies examining the roles of GluN2B-containing NMDARs in LTP have generated mixed results (Shipton and Paulsen, 2014). For example, several lines of lately generated transgenic mice conditionally lacking the GluN2B subunit in the hippocampus only have minor impairment in a weak form of tetanus-induced LTP, but no impairment in a stronger form of LTP in the CA3-CA1 synapse (von Engelhardt et al., 2008; Brigman et al., 2010). Moreover, GluN2B-containing NMDAR antagonists such as Ro25-6981 do not affect high-frequency stimulation-induced LTP in many studies (see Liu et al., 2004; Fox et al., 2006; Papouin et al., 2012 but Bartlett et al., 2007; Berberich et al., 2007). Several factors, including the age of the animals (Ito et al., 1996), LTP induction protocol (von Engelhardt et al., 2008), and neural circuit and brain region (Akashi et al., 2009) may affect the roles of GluN2B-containing NMDAR in LTP induction. Overall, the requirement of the GluN2B subunit for the induction of LTP is likely situation-dependent.

Several factors will need to be considered when interpreting the roles of GluN2B-containing NMDARs in LTP. First, most current studies ignore the contribution of triheteromeric NMDARs, especially those containing both GluN2A and GluN2B subunits. One study provided evidence for the importance of triheteromeric 2A/2B-NMDARs in LTP using carefully designed interrogation of NMDAR subtype functions (Delaney et al., 2012), but exclusive isolation of the triheteromeric NMDAR functions remains challenging with current experimental approaches. Second, transgenic mice studies overexpressing or deleting GluN2B may alter the neuronal development and/or trigger molecular mechanisms to compensate for the changes in the GluN2B subunit expression, thereby misrepresenting the true physiological functions of the subunit (Ito et al., 1996; Tang et al., 1999). Third, multiple forms of NMDAR-dependent LTP may be present (Liu A. et al., 2020), but most experiments testing the roles of GluN2B-containing NMDARs in LTP used a single form of induction protocol limited to the same neural circuit, thus considering LTP as a unified phenomenon. Nevertheless, as illustrated by a thoughtfully designed study using both genetic and pharmacological manipulations (Foster et al., 2010), it is generally accepted that the C-terminal of the GluN2B subunit plays a scaffolding role for recruiting proteins that are critical for LTP induction, whereas the channel function specific for diheteromeric 2B/2B-NMDAR may not be required for the induction of LTP.

### Roles of GluN2B-containing NMDARs in LTD

The NMDAR-dependent form of long-term depression (LTD)-most commonly studied at the hippocampal CA3-CA1 circuit-has been one of the best-characterized mechanisms of activity-dependent reduction in synaptic strength (Collingridge et al., 2010). Increasing amount of evidence suggests an important role of GluN2B-containing NMDARs in the induction of NMDAR-dependent LTD. One of the earliest studies in this field, by using differential activation of NMDAR subpopulations, found that synaptic NMDAR activation mediated LTP, while extrasynaptic NMDAR activation mediated LTD (Lu et al., 2001). Since the GluN2B subunit is predominant expressed in the extrasynaptic location in adult neurons (Stocca and Vicini, 1998), this evidence indirectly supports the importance of GluN2B-containing NMDARs in LTD.

Subsequent pharmacological studies using subtype-specific modulators found that Ro25-6981 was sufficient to block LTD in the CA3-CA1 synapse using acutely prepared brain slices derived from adult animals (see Liu et al., 2004; Fox et al., 2006; Papouin et al., 2012 but Bartlett et al., 2007; Morishita et al., 2007) and *in vivo* using anesthetized animals (Fox et al., 2006; Ge et al., 2010). Notably, both homozygote Cre-dependent GluN2B knockout and heterozygote loss-of-function GluN2B knockin in mice reliably abolish LTD (Ito et al., 1996; Brigman et al., 2010; Shin et al., 2020), providing the strongest evidence for the critical roles of the GluN2B subunit in LTD. Despite the inconsistent results derived from pharmacological studies, a major proportion of evidence, particularly from studies using *in vivo* models, supports an essential role of GluN2B-containing NMDARs in LTD induction.

### Roles of GluN2B-containing NMDARs in learning and memory

GluN2B-containing NMDARs play important roles in a wide range of cognitive functions in rodent models, and this phenomenon is thought to be mediated by their roles in LTP and LTD (von Engelhardt et al., 2008; Brigman et al., 2010). For example, transgenic mice overexpressing the GluN2B subunit in forebrain neurons have improved object recognition memory (Tang et al., 1999), spatial memory (Tang et al., 1999), working memory (Cui et al., 2011), social recognition memory (Jacobs and Tsien, 2012), and motor skill learning (Duan et al., 2018). These enhanced learning abilities may be related to the enhanced LTP in the transgenic mice (Tang et al., 1999; Cui et al., 2011). By contrast, knocking out the GluN2B subunit in mice cortex and hippocampal CA1 regions impaired spatial learning, working memory, and trace fear conditioning (Brigman et al., 2010). Such learning deficits may be related to the impaired LTD (Brigman et al., 2010). Altogether, these pieces of evidence support the indispensable roles of GluN2B-containing NMDARs in many types of learning and memory and the possibility of memory enhancement by enhancing GluN2B-containing NMDAR functions. It should be noted that whether these effects on learning and memory, particularly those observed from the GluN2B subunit-overexpressed transgenic mice, are due to the functional (channel opening) and/or structural (recruiting critical molecules such as CaMKII) roles of GluN2B-containing NMDARs remains to be determined.

Recent behavioral experiments indicate that certain types of cognitive performances, especially those that require flexible adaptations to learned responses, are selectively affected by the manipulation of GluN2B-containing NMDAR functions. Pharmacological inhibition of GluN2B-containing NMDAR function impaired fear memory extinction (Milton et al., 2013), strategy shift during a discrimination task (Dalton et al., 2011), and reversal learning during Morris Water maze (Dong et al., 2013) in rodents, but not their performances on fear conditioning, visual discrimination, and initial spatial learning. Moreover, the impaired behavioral flexibility following GluN2B-antagonism may be mediated by the deficits in LTD, which could be critical for weakening the previously formed associations before new learning can takes place. Indeed, impairment of fear extinction learning in rats by a GluN2B-containing NMDAR antagonist is associated with their impaired LTD in an amygdala circuit (Dalton et al., 2012), while enhancement of spatial reversal learning in mice by NMDAR co-agonist D-serine is associated with their enhanced LTD in the hippocampus (Duffy et al., 2008). Although GluN2B-containing NMDARs have been implicated in other cognitive functions related to memory acquisition (Howland and Cazakoff, 2010; Dong et al., 2012), retrieval (Wong et al., 2007), and forgetting (Sachser et al., 2016), their role in learning tasks that require behavioral flexibility has been the most consistently replicated phenomenon across different behavioral assays and research settings.

### Roles of GluN2B-containing NMDARs in neuronal survival and death

Overactivation of NMDARs during pathological conditions is known to cause excitotoxic neuronal death (known as excitotoxicity), during which different NMDAR subtypes may have distinct, even opposite functions (Wu and Tymianski, 2018; Ge et al., 2020). A large body of literature has suggested that pathological overactivity of GluN2B-containing NMDARs is associated with neuronal death (Ge et al., 2020). For example, neurons expressing chimeric NMDARs with the C-terminal region of the GluN2A subunit replaced by that of the GluN2B subunit experienced increased susceptibility to NMDA-induced cell death, an *in vitro* model of excitotoxic neuronal injury (Martel et al., 2012). By contrast, transgenic mice with parts of the GluN2B C-terminal domain (CTD) region deleted or modified experienced decreased susceptibility to *in vitro* NMDA-induced cell death and an *in vivo* middle cerebral artery occlusion (MCAO) model of stroke (Martel et al., 2012; Vieira et al., 2016; Tang et al., 2018). Furthermore, antagonists that are selective for GluN2B-containing NMDARs, but not those that are preferential for GluN2A-containing NMDARs, protected neurons from excitotoxic death during multiple forms of neuronal injury (DeRidder et al., 2006; Liu et al., 2007; Chen et al., 2008). Although the strict “NMDAR subtype” hypothesis in neuronal survival and death has been challenged by some inconsistent findings (Papouin et al., 2012; Zhou et al., 2013), and by the recent evidence suggesting the roles of triheteromeric 2A/2B-NMDAR and other GluN2-containing NMDAR subtypes in neuronal death (Doyle et al., 2018; Ma et al., 2019), it is generally accepted that GluN2B-containing NMDARs, by coupling to multiple neuronal death signaling complexes, play a dominant role in mediating neuronal toxicity (Wu and Tymianski, 2018; Ge et al., 2020).

## Signaling complexes associated with GluN2B-containing NMDARs

To conserve energy and maximize the efficiency of the cellular processes, neurons often use redundant molecules or signaling pathways to mediate multiple physiological functions, such as synaptic plasticity and neuronal fate, in parallel (see Bartlett and Wang, 2013; Sheng and Ertürk, 2014 for a comprehensive review of these signaling pathways). In most cases, synaptic long-term potentiation shares common signaling mechanisms with neuronal survival signaling, and synaptic long-term depression are mediated by similar processes as neuronal death signaling (Bartlett and Wang, 2013; Sheng and Ertürk, 2014). Largely in consistency with these hypotheses, many GluN2B-dependent signaling pathways regulate both synaptic plasticity and neuronal fate. Here, we will summarize several key protein complexes that associate with the GluN2B subunit and mediate the downstream signaling cascades following GluN2B-containing NMDAR activation.

### GluN2B-CaMKII

**Figure 2:**
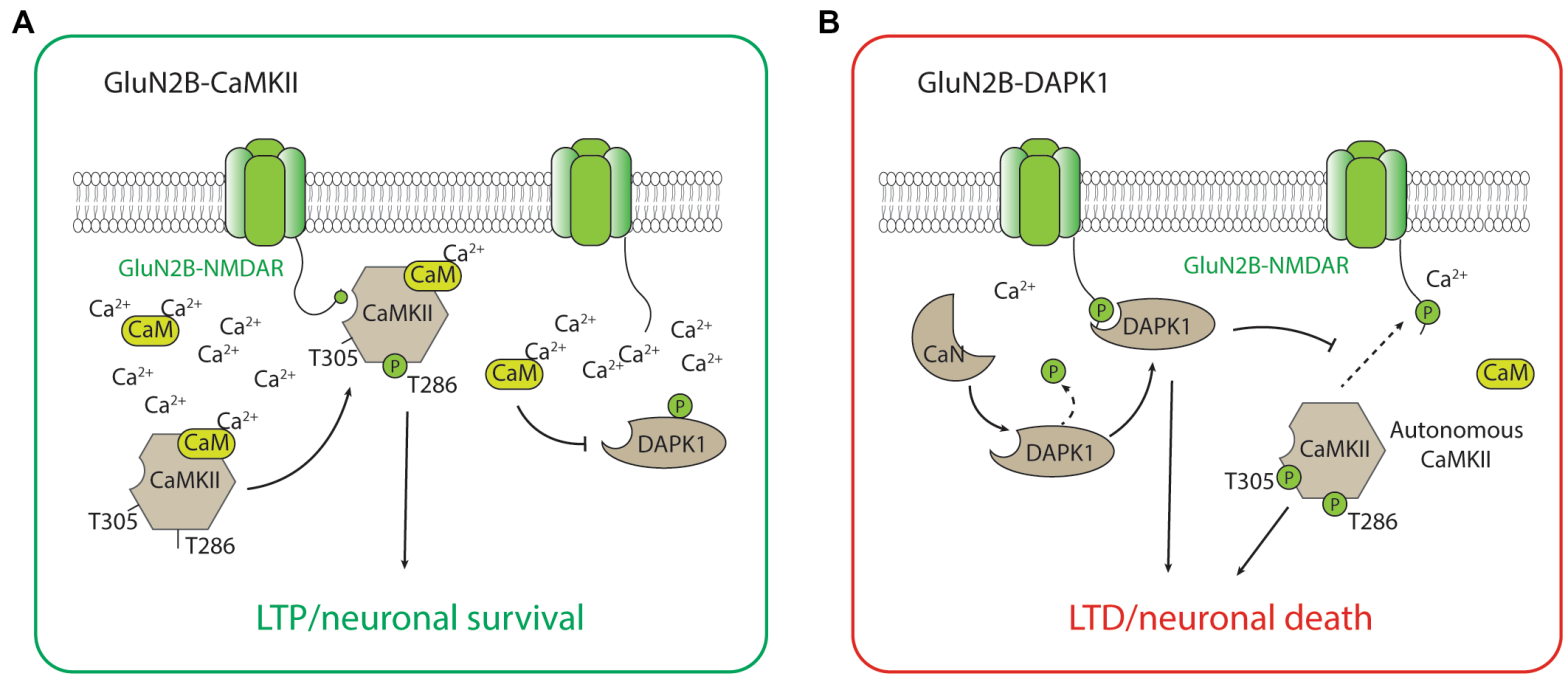
The GluN2B-CaMKII and GluN2B-DAPK1 signaling complexes. **(A)** During high concentration of Ca^2+^, activated CaMKII (with Thr286 autophosphorylation) translocates and forms a complex with GluN2B-containing NMDARs. The association between CaMKII and the GluN2B subunit is required for CaMKII-mediated induction of LTP and the neuronal survival signaling. **(B)** During low to medium concentration of Ca^2+^, calcineurin dephysphorylates and activates DAPK1. The association between DAPK1 and the GluN2B subunit is required for DAPK1-mediated induction of LTD and the neuronal survival signaling. The low Ca2+ condition also favors the autonomous activity of CaMKII (with both Thr286 and Thr305 autophosphorylations), which is involved in the induction of LTD and the neuronal death signaling.

The GluN2B-CaMKII has been the best-characterized GluN2B-dependent signaling complex by far, largely due to the abundance of CaMKII holoenzyme and its importance in synaptic plasticity, especially LTP, and the formation and maintenance of glutamatergic synapses (Lisman et al., 2012; Bayer and Schulman, 2019; Yasuda et al., 2022). During the induction of LTP, a high calcium concentration mediated by the calcium influx through the NMDAR activates calcium calmodulin (CaM), which triggers the CaMKII kinase activity, autophosphorylation of the threonine (Thr)286 residue, and binding to the GluN2B-subunit of the NMDAR (Strack and Colbran, 1998; Bayer et al., 2001; Barria and Malinow, 2005). The binding of CaMKII to GluN2B is critical for inducing the downstream signaling cascades, including the phosphorylation of multiple protein substrates for the CaMKII-dependent potentiation of the synaptic strength (Barria et al., 1997; Tomita et al., 2005; Figure 2). It is important to acknowledge that CaMKII also plays important roles in LTD (Bayer and Schulman, 2019). Notably, the effects of CaMKII on LTD may not be dependent on its binding with GluN2B, but on the autonomous activity of CaMKII (Pi et al., 2010). This autonomous activity is inhibited by a high concentration of CaM but favors a prolonged level of intracellular calcium at low to moderate concentrations (Bayer and Schulman, 2019), and is dependent on the autophosphorylation of Thr305/306 on CaMKII (Pi et al., 2010; Cook et al., 2021; Figure 2). Additionally, LTD stimuli in the excitatory synapse may result in CaMKII translocation to the inhibitory synapse and the subsequent inhibitory LTP (iLTP; Cook et al., 2021). Overall, the roles of CaMKII in bidirectional plasticity are highly consistent with the type of stimulation that is necessary to induce LTP (strong and brief) and LTD (moderate and long) in neurons.

CaMKII has long been implicated in neuronal survival and death, but the direction to which it contributes to neuronal viability has been controversial (Coultrap et al., 2011). Recently, it has been found that a peptide inhibitor tat-CN21, which blocks the autonomous activity of CaMKII, is neuroprotective against glutamate-induced excitotoxicity when applied before or shortly after the insult, whereas CaMKII inhibitor KN-93, which does not block the autonomous activity of CaMKII, has no neuroprotective effect (Vest et al., 2010; Ashpole and Hudmon, 2011). These studies suggest that the autonomous activity of CaMKII, in addition to mediating LTD, may also mediate a critical signaling pathway underlying neuronal death. While the autonomous activity of CaMKII has been increasingly acknowledged as a target for neuroprotection, it is important to note that prolonged inhibition of CaMKII autonomous activity by tat-CN21 for longer than 8 h increased, rather than decreased the susceptibility of neurons to subsequence excitotoxic insults (Ashpole et al., 2012). Moreover, compared to wild-type mice, transgenic mice without CaMKII are more susceptible to ischemic neuron insults, suggesting that the same pathway may also be implicated in neuronal survival signaling (Waxham et al., 1996). Since CaMKII protein may be critical for both neuronal survival and neuronal death, further evidence clarifying the CaMKII-dependent neuronal survival signaling from neuronal death signaling pathways, including the involvement of GluN2B-CaMKII binding in the signaling, will be needed to optimize the CaMKII-based neuroprotective strategy.

### GluN2B-DAPK1

The GluN2B-death-associated protein kinase 1 (DAPK1) complex plays a critical role in mediating downstream signaling cascades following GluN2B-containing NMDAR activation (Wu and Tymianski, 2018; Ge and Wang, 2022). To begin with, the GluN2B-DAPK1 has been discovered as a neuronal death signaling complex which rapidly assemblies upon the activation of extrasynaptic NMDAR during excitotoxic insult (Tu et al., 2010). Transgenic mice lacking the DAPK1 gene or with mutated GluN2B C-terminal region abolishing GluN2B-DAPK1 binding is resistant to ischemic insult during *in vitro* and *in vivo* models of stroke (see Tu et al., 2010; Tang et al., 2018 but McQueen et al., 2017), while interfering the formation of the GluN2B-DAKP1 complex using cell-penetrant peptides Tat-GluN2B_ct_ and Tat-GluN2B_ct_-CTM is neuroprotective against mice models of ischemic stroke (Tu et al., 2010; Fan et al., 2014). Although conflicting evidence regarding the importance of this signaling pathway in neuronal death has been reported, especially regarding the requirement of GluN2B S1303 phosphorylation by DAPK1 during neuronal death signaling (McQueen et al., 2017; Tullis et al., 2021), the GluN2B-DAPK1 complex has still been well-recognized as one of the critical signaling pathways for NMDAR-receptor mediated excitotoxicity and, consequently, an important drug target for treating ischemic stroke.

Other than playing a central role in neuronal death signaling, the GluN2B-DAPK1 complex has recently been found to mediate NMDAR-dependent LTD (Goodell et al., 2017; Figure 2). Interestingly, the signaling of GluN2B-DAPK1 during LTD is dependent on the protein phosphatase calcineurin (Tu et al., 2010; Goodell et al., 2017), and this new evidence is highly coherent with the well-recognized roles of protein phosphatases in LTD. Moreover, DAPK1 competitively prevents the formation of the GluN2B-CaMKII complex, and this process blocks the induction of LTP by CaMKII and favors the induction of LTD (Goodell et al., 2017). The unique roles of DAPK1 in regulating GluN2B-CaMKII complex activity may be critical for determining the direction of synaptic plasticity (Bayer and Schulman, 2019). These studies illustrating the roles of the GluN2B-DAPK1 complex in LTD, together with the recent studies describing the roles of CaMKII in LTD, help provide a coherent explanation regarding the signaling pathways underlying NMDAR-dependent LTP and LTD (Figure 2).

### GluN2B-RasGRF1

The GluN2B-Ras Protein Specific Guanine Nucleotide Releasing Factor 1 (RasGRF1) complex mediates another highly important signaling pathway for the induction of LTD, whereas the GluN2A-RasGRF2 signaling favors the production of LTP (Li et al., 2006; Feig, 2011; Miller et al., 2013). RasGRF1 is a small GTPase exchange factor (GEF) that binds specifically to the C-terminal region of the GluN2B subunit, but not that of the GluN2A or GluN1 subunits (Krapivinsky et al., 2003). The binding of RasGRF1 with GluN2B-containing NMDARs allows it to function as a calcium sensor and an activator of the p38 mitogen-activated protein kinase (MAPK) signaling pathway, which plays critical roles in LTD (Kim et al., 2005; Falcicchia et al., 2020). Interestingly, transgenic mice lacking RasGRF1 are specifically impaired in NMDAR-dependent LTD but not LTP, further supporting that the activation of RasGRF1 is more critical for this form of synaptic plasticity (Li et al., 2006). Besides mediating an important signaling pathway for LTD, some evidence suggests that the GluN2B-RasGRF1 complex may regulate neuronal fate. In comparison with their wild-type counterparts, transgenic mice lacking both RasGRF1 and RasGRF2 are more susceptible to neuronal infarction induced by MCAO (Tian et al., 2004). Moreover, p38 MAPK, the downstream effector of RasGRF1, is critical for mediating neuronal excitotoxicity (Cao et al., 2004). Given that LTD and neuronal death often involve similar signaling cascades, the role of the GluN2B-RasGRF1 complex in neuronal fate, particularly in neuronal death signaling, may warrant further investigation.

### GluN2B-PSD95-nNOS

The GluN2B-postsynaptic density protein 95 (PSD95)-neuronal nitric oxide synthase (nNOS) complex has been an established signaling complex mediating neuronal death and an important drug target for neuroprotection (Sattler et al., 1999; Wu and Tymianski, 2018; Ge and Wang, 2022). Multiple drugs or interfering peptides, including NA-1 (also known as Tat-NR2B^C9^; Aarts et al., 2002), Tat-N-dimer (Bach et al., 2012), and ZL006 (Zhou et al., 2010), were designed to disrupt the formation of this protein complex during neuronal injury, and these therapeutics achieved neuroprotection in rodent models of ischemic stroke (Zhou et al., 2010; Bach et al., 2012), traumatic brain injury (Qu et al., 2020), and epilepsy (Colciaghi et al., 2019). Notably, in a phase III clinical trial (NCT02930018), NA-1 effectively improved neurological outcomes and reduced the brain infarct volume in ischemic stroke patients undergoing endovascular treatment (Hill et al., 2020). This clinical evidence further validates the GluN2B-PSD95-nNOS complex as an important therapeutic target for neuroprotection and a key mediator of neuronal death signaling. Limited evidence also supports the role of GluN2B-PSD95-nNOS in synaptic plasticity. For example, disrupting this protein complex with ZL006 impaired LTP that was measured from the amygdala in rat brain slices (Li et al., 2018). Nevertheless, the definitive role of this protein complex in bidirectional synaptic plasticity has yet to be verified. Given that several other neuronal death signaling complexes have been implicated in synaptic depression, it may be worthwhile to further explore the roles of the GluN2B-PSD95-nNOS complex in LTD of the glutamatergic synapses.

## The metabotropic function of GluN2B-containing NMDARs

While most of the signaling complexes associated with GluN2B-containing NMDARs are activated by calcium-dependent mechanisms, some investigators have found that GluN2B-containing NMDAR can mediate downstream signaling through a channel function-independent mechanism (Dore et al., 2016). It has been demonstrated that the glutamate binding to the NMDAR, without the channel opening, is sufficient to induce LTD (see Nabavi et al., 2013 but Babiec et al., 2014). This calcium-independent, metabotropic function of NMDARs may be predominantly mediated by GluN2B-containing NMDARs (Tamburri et al., 2013), although one study suggested that the GluN2A and GluN2B subunit contributed equally to this form of LTD (Wong and Gray, 2018). Moreover, the metabotropic function of GluN2B-containing NMDARs has been shown to mediate structural LTD during amyloid beta-induced synaptic loss (Kessels et al., 2013; Stein et al., 2015). In contrast to the widely accepted hypothesis that ion flux through the NMDARs is required for LTD, accumulating evidence suggests that the metabotropic function of NMDARs is sufficient for both structural and functional LTD.

Given that structural LTD involving synaptic loss and neuronal death are often mediated by similar molecular mechanisms (Sheng and Ertürk, 2014), it is reasonable to hypothesize that the metabotropic function of NMDARs may also be critical for neuronal death signaling. Indeed, NMDARs form a metabotropic signaling-dependent neuronal death signaling complex with the pannexin-1 (Panx1) channel. Blocking the channel function or dissociating this protein complex using an interfering peptide is neuronal protective against oxygen–glucose deprivation (OGD)-induced neuronal death, an *in vitro* model of ischemic stroke (Weilinger et al., 2016). Given that GluN2B-containing NMDAR is a critical NMDAR subtype mediating neuronal death signaling, it would be interesting to determine the contribution of the GluN2B subunit vs. other GluN2 subunits in the metabotropic NMDAR function-related neuronal death. Future studies should also illustrate the relative contribution of metabotropic and ionotropic NMDAR functions in neuronal death signaling using multiple neuronal toxicity models.

## GluN2B hypofunction in neurological diseases

In addition to regulating synaptic plasticity and neuronal fate in mature neurons, GluN2B-containing NMDARs have been recognized to play critical roles in neuronal development (reviewed in Paoletti et al., 2013). Consequently, heterozygote loss of function of the GluN2B subunit has been associated with autism spectrum disorder (ASD) and related developmental disorders in both human patients and animal models (Liu et al., 2017; Vyklicky et al., 2018; Sceniak et al., 2019; Wang et al., 2022). Consistent with these results, several genetic mice models of ASD and ASD-related developmental disabilities all exhibit hypofunction of GluN2B (Peça et al., 2011; Li et al., 2015; Toft et al., 2016), suggesting the deficit of GluN2B function as a common postsynaptic mechanism that is associated with multiple neurodevelopmental diseases. In accordance with this evidence, early administration of an NMDAR co-agonist D-cycloserine in transgenic mice harboring an ASD-risk GluN2B mutation corrected the ASD-related synaptic deficits and behavioral abnormalities in adult mutant mice (Shin et al., 2020). This evidence indicates that correcting GluN2B-containing NMDAR hypofunction during the developmental period may be a viable strategy for treating ASD-related neurodevelopmental disorders.

In consistency with the critical role of GluN2B-containing NMDARs in synaptic plasticity and cognition, GluN2B hypofunction has been commonly associated with both intellectual disabilities in children and cognitive deficits during aging (Wang et al., 2014; Vyklicky et al., 2018). Pathogenic GluN2B variants have been nearly invariably associated with intellectual disability, which is diagnosed by severely low intelligence quotient (IQ) and limitation in daily activities (García-Recio et al., 2021). Moreover, the GluN2B subunit expression experiences age-dependent reduction. This decreased protein expression is conserved across species and is correlated with reduced cognitive performances (Wang et al., 2014; Pegasiou et al., 2020). Interestingly, both germline and virus-mediated overexpression of the GluN2B subunit enhanced learning and memory performances in aged mice, especially in the retention of long-term spatial memory (Cao et al., 2007; Brim et al., 2013). Together, these data indicate the critical role of GluN2B hypofunction in both normal and pathological memory decline, suggesting the potential for boosting GluN2B-NMDAR function for cognitive enhancement.

NMDAR hypofunction has been widely recognized to play a critical role in schizophrenia, but the contribution of GluN2B hypofunction to schizophrenia has yet to be determined (Pratt et al., 2012). Interestingly, impaired behavioral flexibility has been the most consistent cognitive deficit in schizophrenic patients (Floresco et al., 2009). This finding, together with the consistent role of GluN2B-containing NMDAR in tasks that require behavioral flexibility in rodent studies (Dalton et al., 2011; Marquardt et al., 2019), indicates the potential role of GluN2B-containing NMDAR in the cognitive deficits of schizophrenia. Nevertheless, a recent large-scale genetic study has identified the GluN2A, but not the GluN2B subunit as a key schizophrenia risk gene (Singh et al., 2022), suggesting a superior role of the GluN2A subunit in the pathogenesis of schizophrenia and a target for disease intervention.

## GluN2B hyperfunction in neurological diseases

Overactivation of GluN2B-containing NMDARs is a key pathogenetic mechanism for neuronal excitotoxicity, which may play critical roles in neuronal injury during stroke, traumatic brain injury, and epilepsy (Wu and Tymianski, 2018; Ge et al., 2020). In preclinical animal models, blocking GluN2B-containing NMDAR currents is effective against ischemic neuronal death *in vitro* and *in vivo* (DeRidder et al., 2006; Liu et al., 2007; Chen et al., 2008); however, the bench-to-bedside translation of these therapeutics has proven to be challenging, likely due to them causing unwanted side effects and having short therapeutic windows (Ge et al., 2020). Recently, there has been a strategic shift toward targeting the downstream signaling complex associated with the GluN2B subunit, with the hope of improving the therapeutic time window (Sun et al., 2015). Moreover, a recent study investigating the time course of GluN2B-containing NMDAR activation during stroke has suggested that the initial overactivation of GluN2B may be followed by a prolonged hypoactivity of GluN2B-containing NMDAR during stroke recovery (Liu et al., 2010). In line with these results, enhancing NMDAR activity using D-cycloserine post-stroke has been shown to promote functional recovery in rats after MCAO (Dhawan et al., 2011). Therefore, the timing of GluN2B-containing NMDAR activation during ischemic stroke needs to be carefully investigated. The activation, rather than inhibition of GluN2B-containing NMDAR activity may be an effective treatment strategy during stroke recovery.

## GluN2B dysregulation in other neurological diseases

GluN2B-containing NMDARs and their associated protein complexes may be critical targets for treating major depressive disorder (MDD; Paoletti et al., 2013; Ge and Wang, 2022). The hypothesis for targeting glutamate receptors in MDD derived at least partially from the discovery and approval of ketamine, an NMDAR channel blocker, as a rapid-onset antidepressant used for treatment-resistant MDD (McGirr et al., 2015). Recently, several studies focusing on ketamine’s mechanisms of action have demonstrated that its antagonism on the GluN2B-containing subtype of NMDARs is required for its antidepressant effects (Miller et al., 2014; Gerhard et al., 2020; Pothula et al., 2021a). These studies strongly support the possibility of targeting NMDARs, especially the GluN2B-containing subtype, for the treatment of MDD. It has been found subsequently that both NMDAR inhibitors and positive allosteric modulators (PAM) may produce antidepressant-like effects in rodent models (Li et al., 2011, 2021; Pothula et al., 2021a,b), raising the question regarding the direction of GluN2B-containing NMDAR dysregulation during MDD. One hypothesis is that the antidepressant effects of NMDAR antagonists and PAMs are mediated by different cell types; NMDAR antagonists inhibited the GluN2B-containing NMDAR function on interneurons (Pothula et al., 2021a), whereas NMDAR PAMs enhanced the GluN2B-containing NMDAR function on pyramidal neurons (Pothula et al., 2021b). Both mechanisms were implicated in the antidepressant effects of these NMDAR-targeting drugs. Overall, targeting GluN2B-containing NMDAR may produce ketamine-like rapid antidepressant effects and may lead to the development of a new class of antidepressant drugs for a sizeable proportion of patients who do not respond to the standard-of-care pharmacotherapies.

Dysregulated fear learning has been a key characteristic in patients suffering from post-traumatic stress disorder (PTSD). Since GluN2B-containing NMDARs play critical roles in fear memory stability (Mamou et al., 2006), fear extinction (Dalton et al., 2012), and fear memory generalization (Asim et al., 2020), dysfunction of GluN2B-containing NMDARs may underlie the symptoms of PTSD. Indeed, the GluN2B subunit expression in mice brains is elevated following a strong fear experience, and normalizing the GluN2B-containing NMDAR function may be critical for preventing the generalization of the learned fear response (Asim et al., 2020). Moreover, through a GluN2B-containing NMDAR-dependent mechanism, NMDAR PAM NYX-783 reduced the spontaneous recovery of fear following fear extinction training in rats (Lee et al., 2022). This drug has shown efficacy in treating patients with PTSD in a phase II clinical trial (NCT04044664). Although a direct link between dysfunction in synaptic plasticity and PTSD has not been rigorously established, current evidence suggests that targeting GluN2B-containing NMDAR-dependent plasticity may be an effective way for treating PTSD.

Neuronal degeneration or excitotoxicity, mediated by the overactivation of GluN2B-containing NMDARs, has been a common feature among multiple neurodegenerative diseases, including Alzheimer’s disease, Parkinson’s disease, and Huntington’s disease (Paoletti et al., 2013). Based on the roles of GluN2B-containing NMDAR in neuronal death signaling, inhibiting extrasynaptic, GluN2B-containing NMDAR function has frequently been tested as a strategy for preventing neuronal degeneration (Hardingham and Bading, 2010). However, the translation of these therapeutics to the clinic has mostly failed, raising the need for alternative approaches to reverse the synaptic dysfunction in these diseases (Liu W. et al., 2020). Recently, there has been a surge in developing NMDAR PAMs for improving synaptic plasticity, thus the cognitive symptoms in multiple neurodegenerative diseases (Hill et al., 2022; Lee et al., 2022). One of these drugs, known as SAGE-718, is being evaluated in phase II clinical trials for cognitive symptoms in Alzheimer’s disease (NCT05619692), Parkinson’s disease (NCT05318937), and Huntington’s disease (NCT05107128). The renewed interest in targeting NMDAR dysfunction may be critical for improving the functional outcomes in patients with neurodegenerative disease, especially considering the slow progress in the development of disease-modifying therapeutics in the past decades.

## Future directions

Understanding the pharmacological properties of GluN2B-containing NMDARs and having the tools to manipulate their functions, accordingly, has led to important discoveries related to these receptors (Paoletti et al., 2013; Ge et al., 2020). On the flip side, the lack of experimental techniques to specifically manipulate some receptor subpopulations, especially the triheteromeric GluN2B-containing NMDARs, has limited our understanding of their physiological functions. Although some recent studies have attempted to develop novel methods to investigate triheteromeric NMDARs (Stroebel et al., 2014), or to specifically target diheteromeric NMDARs (Khatri et al., 2014), further research is needed to develop more sophisticated tools to understand the full diversity of GluN2B-containing NMDAR functions.

Several GluN2B-containing NMDAR-associated signaling complexes have been identified, many of which mediate the crosstalk between the GluN2B subunit-mediated synaptic plasticity and neuronal fate (Wu and Tymianski, 2018; Ge and Wang, 2022); however, much less is known about the interaction between these signaling complexes and the relative contribution of each to these physiological functions. Future studies may use a network, multiomics approach to generate more coherent and unbiased representations of the GluN2B-dependent signaling pathways during synaptic plasticity and neuronal survival and death signaling. Moreover, with the increased appreciation of the metabotropic function of GluN2B-containing NMDARs, it would be critical to isolate the signaling pathways mediated by the ionotropic and metabotropic function of the receptor and describe the potential interactions between them.

Most previous studies have focused on inhibiting GluN2B-containing NMDAR function for treating neuropsychiatric diseases, but, recently, there has been a paradigm shift in the therapeutic strategy toward enhancing GluN2B-containing NMDAR function, particularly in an allosteric manner. This new approach has been investigated for improving stroke recovery, major depressive disorder, PTSD, and cognitive symptoms in neurodegenerative diseases (Dhawan et al., 2011; Pothula et al., 2021b; Lee et al., 2022). Future studies should further pinpoint the GluN2B-containing NMDAR dysfunction in neurological disorders to validate this approach. For instance, it would be critical to further understand the temporal dynamics, cell types, and brain regions that are involved in GluN2B-containing NMDAR dysfunction. With the recent advancement of NMDAR PAMs in clinical studies, future research may develop subtype-specific PAMs targeting GluN2B-containing NMDARs with potentially improved therapeutic profile for neurological diseases. Given the accumulating structural biology, preclinical, and clinical data for therapeutics targeting NMDARs, using computer-assisted, structural-based drug design methods combined with machine learning approaches may maximize the success rates of such drug discovery projects.

## Author contributions

All authors listed have made a substantial, direct, and intellectual contribution to the work and approved it for publication.

## Funding

This work was supported by CIHR Canada Foundation grant (FDN-154286).

## Conflict of interest

The authors declare that the research was conducted in the absence of any commercial or financial relationships that could be construed as a potential conflict of interest.

## Publisher’s note

All claims expressed in this article are solely those of the authors and do not necessarily represent those of their affiliated organizations, or those of the publisher, the editors and the reviewers. Any product that may be evaluated in this article, or claim that may be made by its manufacturer, is not guaranteed or endorsed by the publisher.
